# Localization of Engineered Vasculature within 3D Tissue Constructs

**DOI:** 10.3389/fbioe.2018.00002

**Published:** 2018-01-22

**Authors:** Shira Landau, Shaowei Guo, Shulamit Levenberg

**Affiliations:** ^1^Department of Biomedical Engineering, Technion – Israel Institute of Technology, Haifa, Israel

**Keywords:** blood vessels, angiogenesis, 3D scaffolds, pericytes, migration

## Abstract

Today, *in vitro* vessel network systems frequently serve as models for investigating cellular and functional mechanisms underlying angiogenesis and vasculogenesis. Understanding the cues triggering the observed cell migration, organization, and differentiation, as well as the time frame of these processes, can improve the design of engineered microvasculature. Here, we present first evidence of the migration of endothelial cells into the depths of the scaffold, where they formed blood vessels surrounded by extracellular matrix and supporting cells. The supporting cells presented localization-dependent phenotypes, where cells adjacent to blood vessels displayed a more mature phenotype, with smooth muscle cell characteristics, whereas cells on the scaffold surface showed a pericyte-like phenotype. Yes-associated protein (YAP), a transcription activator of genes involved in cell proliferation and tissue growth, displayed spatially dependent expression, with cells on the surface showing more nuclear YAP than cells situated deeper within the scaffold.

## Introduction

Blood vessel growth within engineered tissues is a critical factor in overcoming insufficient blood perfusion in implants (Shandalov et al., 2014; Mirabella et al., 2017). Endothelial and supporting cell cocultures are often used to generate such blood vessels (Koike et al., 2004; Koffler et al., 2011; Freiman et al., 2016; Landau et al., 2017). While this cell combination spontaneously forms micro-vasculature, the mechanism controlling the process is not well understood.

Endothelial cell migration occurring during angiogenesis is regulated by (1) chemotaxis: the migration of cells in the direction of soluble chemoattractants gradient, (2) haptotaxis: the migration of cells in the direction of tied ligands, and (3) mechanotaxis: the migration of cells in response to mechanical cues. In addition, cell mediated degradation of the extracellular matrix also plays an important role in endothelial cell migration (Lamalice et al., 2007). Distinct differences in vessel network environments exist in the body. For example, single or multiple layers of vascular smooth muscle cells (vSMCs) surround large vessels as opposed to intermediate-sized vessels which are surrounded by mural cells that have shared properties of both vSMCs and pericytes, which in many cases, serve as vSMC progenitors (Holger and Christer Betsholtz, 2003; Bergers and Song, 2005; Volz et al., 2015). Hence, cellular phenotype is influenced by the cell’s spatial localization. Thus we hypothesized that during the process of blood vessel formation within engineered constructs, endothelial cells migrate in response to stimulation cues and that the phenotype of supporting cells shifts, in accordance with their spatial localization.

This study was designed to assess ECs migration during angiogenesis and the location-specific characteristics of the cells composing the forming vessels within different three-dimensional (3D) constructs. While previous studies examined the dynamics of vessel network formation *in vitro* (Blinder et al., 2015; Freiman et al., 2016), the present experimental setup aimed to identify the effect of cellular localization at different layers of the scaffold on vessel formation metrics such as migration, proliferation, protein expression and differentiation of endothelial and supporting cells. Understanding these processes can aid in designing improved vascularized constructs, which will enhance graft integration upon implantation.

## Materials and Methods

### Scaffolds

Gelfoam scaffolds were purchased from Pfizer and were cut into pieces 1 cm long, 0.5 cm wide, and 1.5 mm thick pieces. PLLA/PLGA scaffolds were prepared as followed: 0.4 g NaCl particles were covered with 0.24 ml PLLA/PLGA solution, which was dissolved in chloroform, and evaporated overnight. Salt was then leached out by four washes, leaving behind pores within the scaffold. Scaffolds were then cut into 6 mm diameter circles with the thickness of 0.8 mm. Fibrin gel was obtained by mixing thrombin (20 U/ml, Sigma-Aldrich) with fibrinogen (15 mg/ml, Sigma-Aldrich).

### Cell Culture

Human adipose microvascular endothelial cells (HAMECs; ScienceCell), lentivirally transduced with ZsGreen fluorescent protein, were grown in endothelial cell medium (ScienceCell), supplemented with 5% FBS (ScienceCell), and endothelial cell growth supplement (ScienceCell), and used for five to nine passages. Neonatal normal human dermal fibroblasts (HNDFs) (Lonza Walkersville Inc.) were grown in Dulbecco’s modified Eagle medium (DMEM) (Gibco), supplemented with 10% FBS (HyClone), 1% non-essential amino acids (NEAAs), 0.2% β-mercaptoethanol (Sigma-Aldrich), and 1% penicillin–streptomycin solution (PEN STREP) (Biological Industries). Dental pulp stem cells (DPSCs) (Lonza) were cultured in low glucose DMEM (Gibco), supplemented with 10% FBS (HyClone), 1% NEAAs, 1% GlutaMAX (Gibco), and 1% penicillin–streptomycin-nystatin solution (Biological Industries). Three dimensional, vascularized constructs were obtained by coseeding endothelial cells (3 × 10^5^ cells) and DPSCs (9 × 10^5^) or fibroblasts (0.6 × 10^5^ cells) in 20 µl medium on the gelfoam or in 7 µl fibrin on the PLLA/PLGA scaffolds followed by incubation of 30 min, before addition of medium. For the mitomycin experiments, 5 ml mitomycin (Sigma-Aldrich) was applied to the fibroblast cells 2 h before seeding, and washed twice with PBS.

### Lentivirus Packaging and HAMEC Transduction with ZsGreen and dTomato Fluorescent Proteins

The Lenti-X HTX Packaging System (Clontech, a fourth generation) was used to generate recombinant, replication-incompetent VSV-G pseudotyped lentiviruses, according to the manufacturer’s instructions. Transfection of the expression vector into the Lenti-X 293 T packaging cells (Clontech) was performed in a Lenti-X HTX Packaging mix. The supernatants of transfected packaging cells were collected 72 h later and filtered through a 45-μm filter before being added to the HAMECs, with 6 mg/ml polybrene (Sigma-Aldrich). The transduction medium was replaced by culture medium 24 h thereafter, and cells were then cultured for 72 h to allow gene product accumulation in the cells. Cells were then selected using 1 mg/ml puromycin (Takara Bio Company).

### Whole-Mount and Cryosection Immunofluorescence Staining

Whole constructs were fixated in paraformaldehyde (4%), for 20 min, and then permeabilized with 0.3% Triton X-100 (Bio Lab Ltd.), for 10 min. Constructs were then washed with PBS and immersed overnight in BSA solution (5%; Millipore). Samples were then incubated with goat antihuman VE-cadherin (1:100; Santa Cruz), mouse antihuman Yes-associated protein (YAP) (1:100; Santa Cruz), mouse antihuman NG2 (1:100; Santa Cruz), rabbit antihuman vWF (1:150; Abcam), rabbit antihuman β-catenin (1:100; Sigma-Aldrich), or mouse antihuman Ki67 (1:20, DAKO) antibodies, overnight, at 4°C. Constructs were then treated with Cy3-labeled (1:100; Jackson Immunoresearch Laboratory), Cy5-labeled (1:100; Jackson Immunoresearch Laboratory), or Alexa-488 (1:400; ThermoFisher Scientific) antibodies, mixed with DAPI (1:1000; Sigma-Aldrich), for 2 h, at room temperature. For the phalloidin staining, constructs were treated with FITC phalloidin (1:100; Sigma-Aldrich) and DAPI for 20 min. For the mitomycin experiment, mitomycin-treated cells and control cells without mitomycin were fixated in paraformaldehyde (4%), for 20 min on day 10 of culture, and then incubated in a 30% (wt/vol) sucrose solution overnight, embedded in optimal cutting temperature compound (Tissue-Tek) and frozen for subsequent cryosectioning (5–20 µm). Standard protocols were used for H&E and Masson’s trichrome staining of the sections.

### Construct Imaging and Quantification of the Images

Whole vascularized constructs were imaged using a confocal microscope (LSM700, Zeiss), equipped with 20× and 63× oil immersion lenses. Three-dimensional images were projected into 2D images, using maximum intensity projection, and the stacks were then separated into three main regions: the surface (0–10 µm), middle (10–20 µm), and deeper (20 μm-end) areas of the scaffold. Confocal images were then analyzed using a self-written algorithm in MATLAB, for ki67 quantification: images were transformed into binary images and pixel density was calculated. Vessel quality was determined using a self-written algorithm in MATLAB: images were transformed into binary images and the eccentricity parameter, an indicator of the deviation of an element from circularity, was calculated for each separate element in the image. Elongated vessels received a higher eccentricity score, whereas, cells clusters and disrupted vessels, which are more circular, receive lower eccentricity scores. Imaris software (BITPLANE) was used to detect YAP and β-catenin localization through the 3D image.

### Statistical Analysis

Presented data include the mean ± SD. Two-way analysis of variance was performed to examine the influence of two independent categorical variables, followed by Bonferroni’s multiple comparison tests. Results were considered significant for *p* < 0.05. Statistical analysis was performed using a computerized statistical program (GraphPad Software). Experiments were repeated three times.

## Results

### Vessel Formation within 3D Constructs

**Figure 1:**
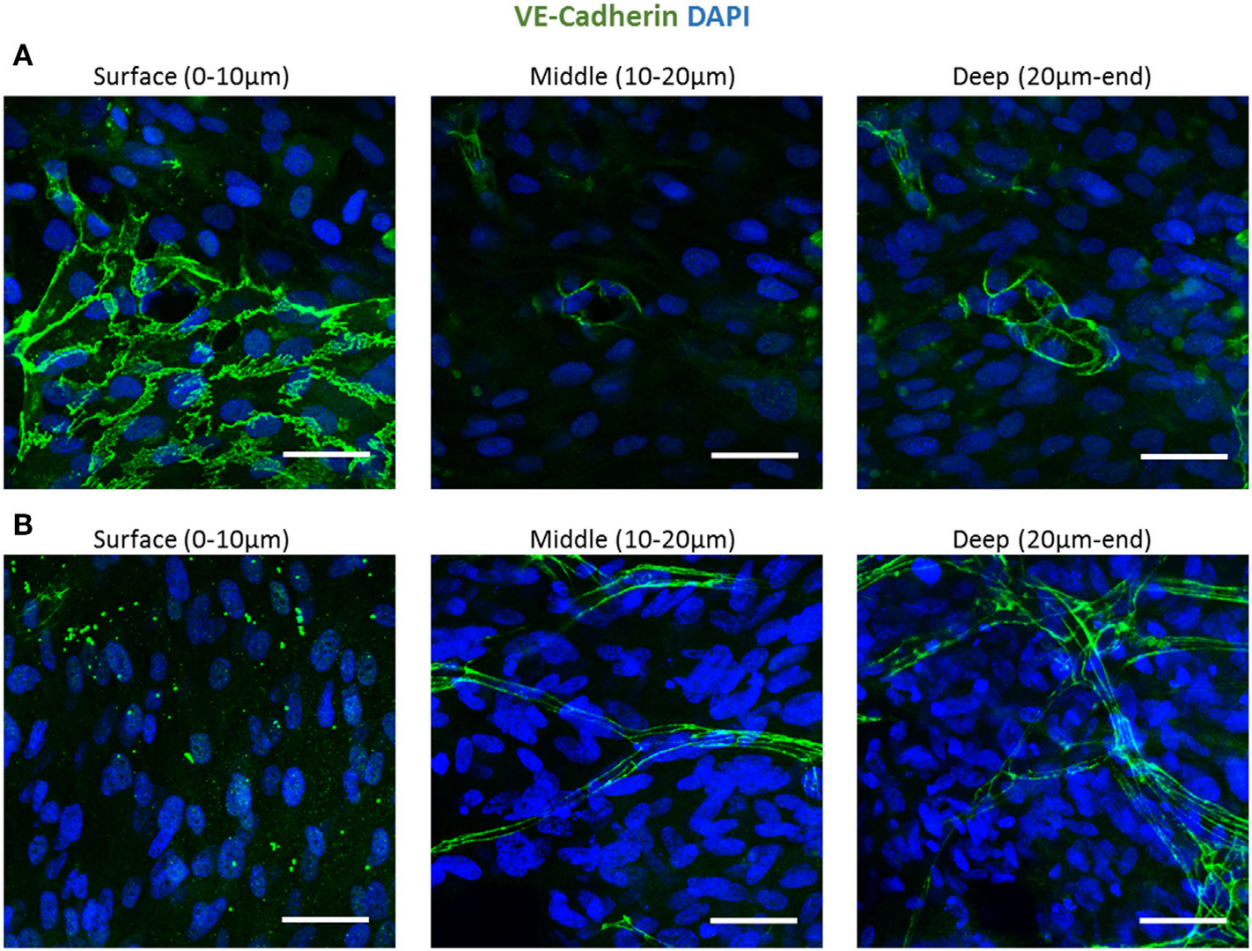
Endothelial cell configuration throughout a 3D gelfoam scaffold. ECs and fibroblasts were seeded into a gelfoam scaffold and were fixated and then stained for VE-cadherin (green) and with DAPI nuclear stain (blue). Images demonstrate the endothelial cell morphology at different depths of the scaffold **(A)** after 7 days in culture. ECs on the scaffold surface formed a sheet and vessels began to form in the depths of the scaffold. **(B)** On day 14 of culturing, a fully developed vessel network was observed in the scaffold depths. Scale bar indicates 50 µm.

**Figure 2:**
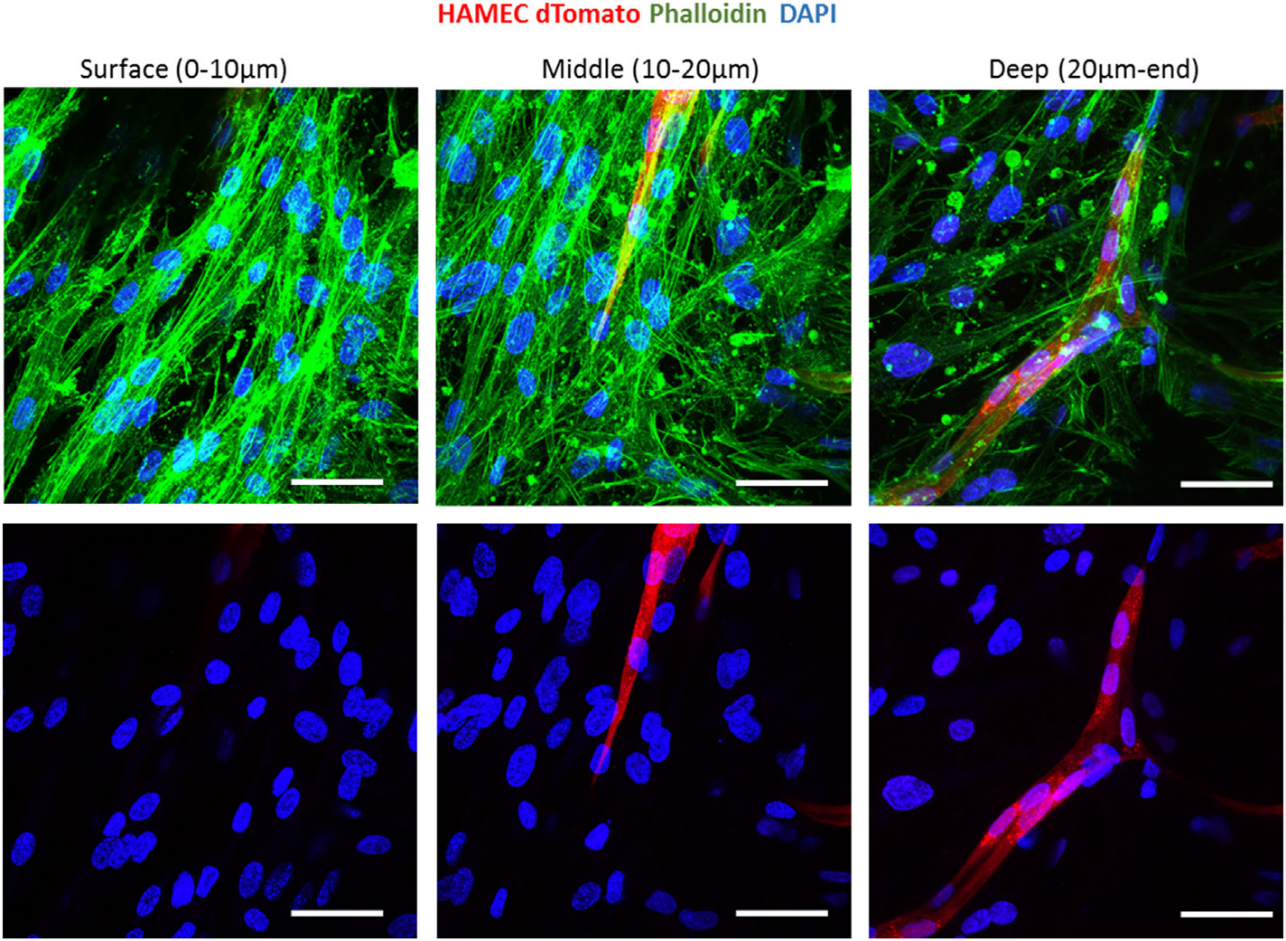
Vessel localization is not limited to a certain scaffold or cell type. Vessel morphogenesis within a PLLA-PLGA scaffold embedded with fibrin, at different depths of the scaffold. Cells were stained with phalloidin (green) and DAPI (blue). ECs were marked with dTomato (red). Scale bar indicates 50 µm.

To follow vessel formation dynamics within 3D structures, a coculture of HAMECs and HNDFs was seeded into a gelfoam scaffold. After 7 days of culturing, cells were stained for VE-cadherin and were imaged using a confocal microscope. A sheet-like structure of an endothelial cell monolayer was observed on the surface of the scaffold, whereas in the scaffolds depth, cells began to form microvessels (Figure 1A). On day 14 postseeding, the endothelial cell monolayer structure was no longer observed and only microvessels were observed in the scaffolds depth; 3D confocal imaging showed that these vessels were lumenalized. In addition, at the same time point, a dense fibroblast layer was apparent on the scaffold surface and around the vessels (Figure 1B; Figures S1 and S2 in Supplementary Material). To confirm that this phenomenon was not limited to a certain scaffold or cell type, dTomato HAMECs were seeded with DPSCs into a PLLA/PLGA scaffold, which was then stained with DAPI and phalloidin. DPSCs were mainly observed on the scaffold surface and endothelial micro-vessels were seen within the scaffold depth (Figure 2).

### PHD2 Expression throughout the Scaffold

**Figure 3:**
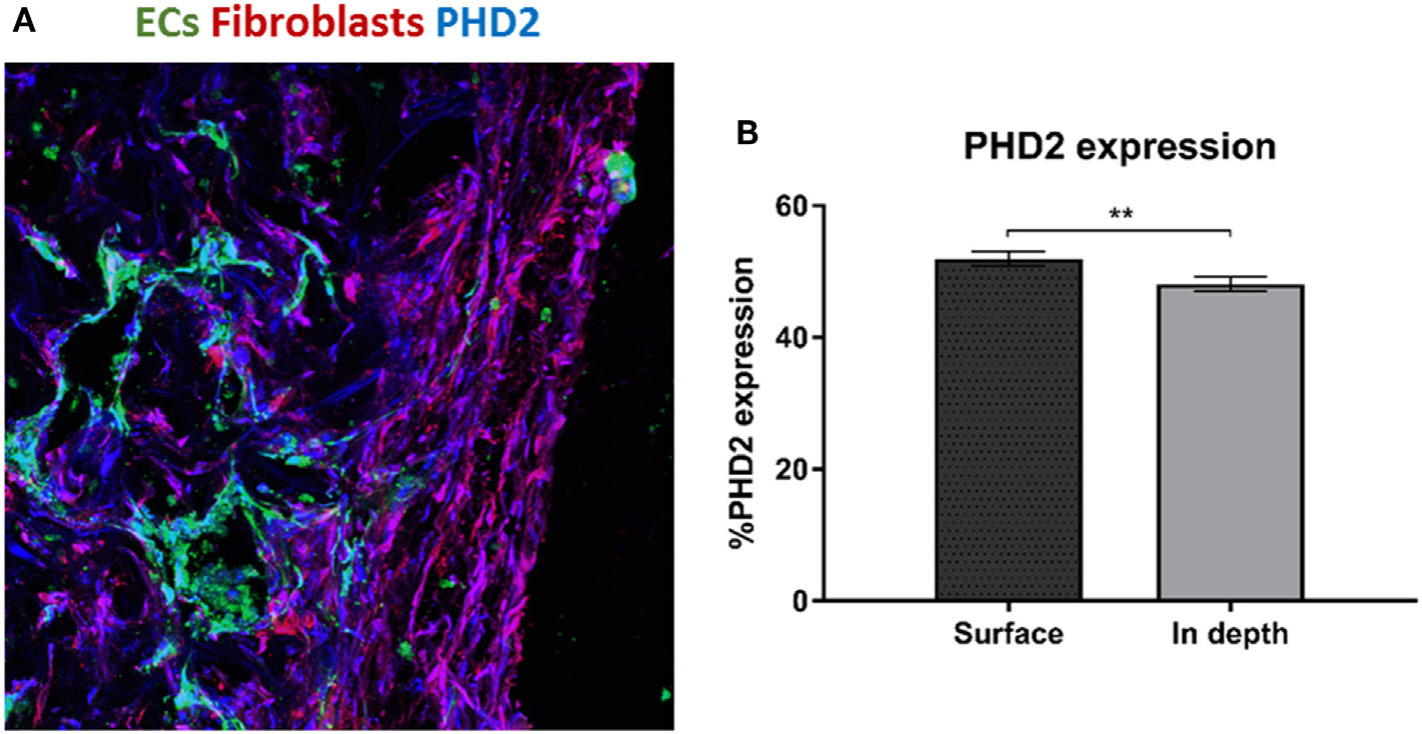
PHD2 expression throughout the scaffold. **(A)** Staining of a transversed cryosection with PHD2 (blue), endothelial cells are marked in green and fibroblasts in red. **(B)** Quantification of PHD2 expression at the scaffold surface and in the scaffold depth. (*n* = 3) **P-value <0.01.

We hypothesize that EC migration toward the scaffolds interior might be due to hypoxia. It is recognized that secretion of VEGF increases in hypoxic conditions (Shweiki et al., 1992) which consequently triggers EC migration. Transversed cryosections were stained with PHD2 (prolyl hydroxylase domain 2), which flags hypoxia inducing factor alpha subunits for ubiquitin-proteasome degradation under normoxic conditions (Carmeliet and Jain, 2011). PHD2 expression was higher at the scaffold surface as compared to the scaffolds depth (Figures 3A,B). Hence, the increasing hypoxic conditions at the scaffold depths may have triggered the ECs to form vessels there.

### Fibroblast Localization and Proliferation throughout the Scaffold

**Figure 4:**
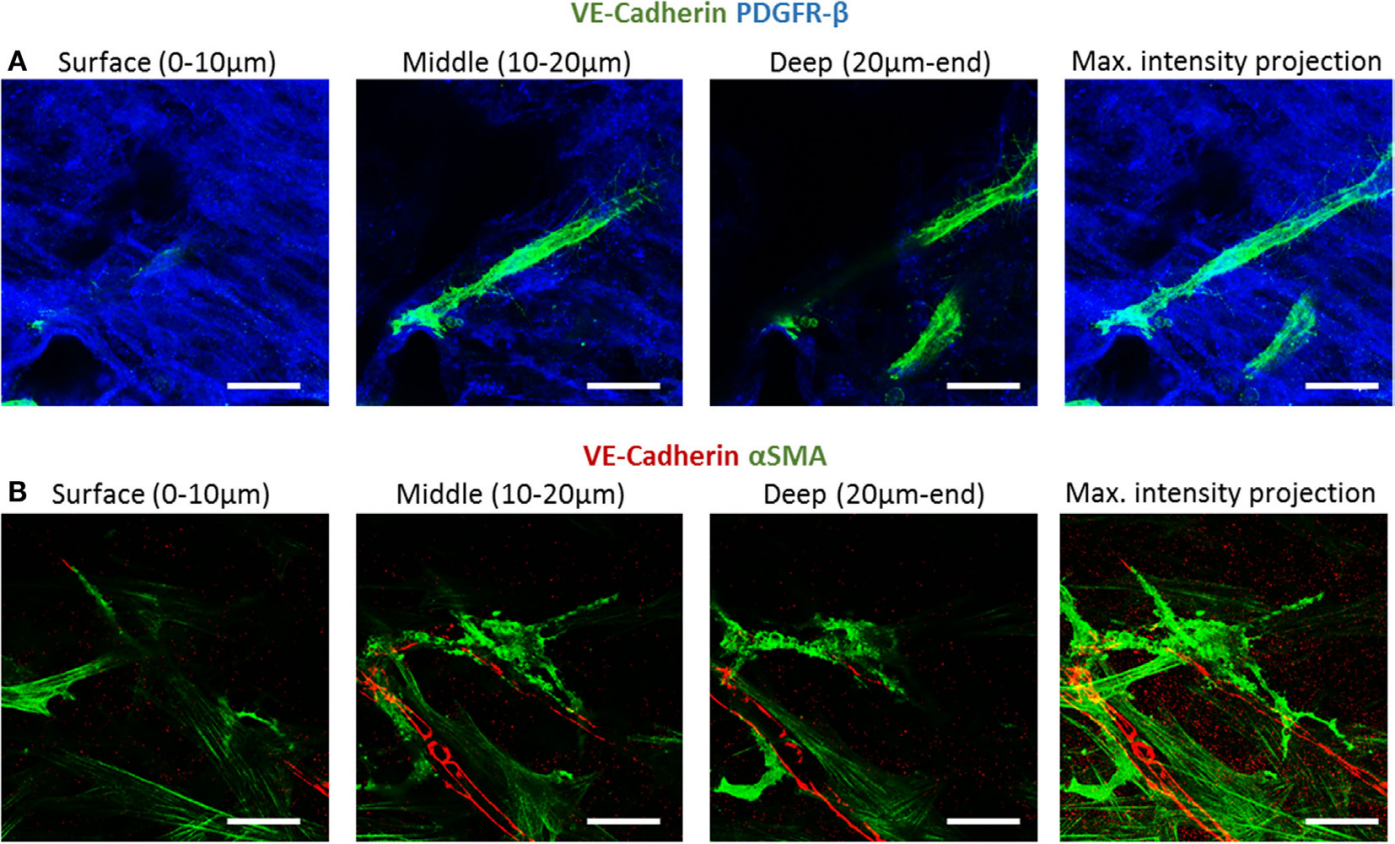
PDGFRβ and αSMA expression across the scaffold. Gelfoam scaffolds seeded with ECs and fibroblast, were cultured for 14 days and then stained for: **(A)** CD31 (green) and PDGFRβ (blue) or **(B)** CD31 (red) and αSMA (green). Expression patterns were assessed at various depths of the scaffolds. Scale bar indicates 50 µm.

**Figure 5:**
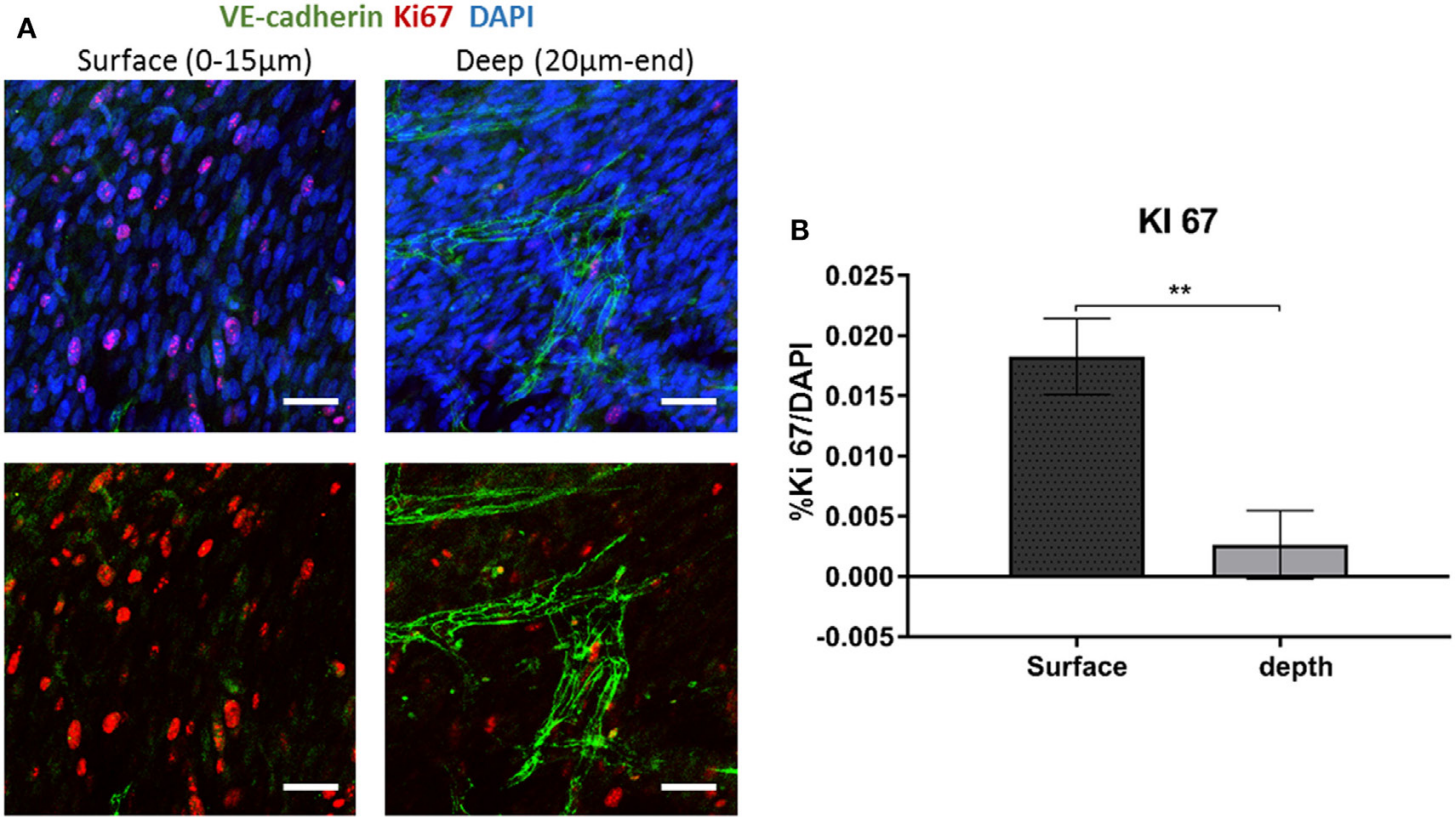
Ki67 expression across the scaffold. **(A)** Gelfoam scaffolds were seeded with ECs and fibroblast, cultured for 14 days, and then stained for Ki67 (red) and VE-cadherin (green). **(B)** Expression patterns were assessed at various depths of the scaffolds. The percentage of Ki67-positive cell on the surface and the depth of the scaffold was quantified. Scale bar indicates 50 µm. (*n* = 3) **P-value <0.01.

To examine fibroblast characteristics in the 3D constructs, a HAMEC and HNDF coculture was seeded into the gelfoam scaffold and grown for 14 days, fixated and stained for CD31, to mark the ECs, and for PDGFRβ and αSMA, to mark the pericytes; expression of the two proteins has been shown to increase during the recruitment of pericytes to stabilize the forming vessels (Welti et al., 2013). Confocal imaging revealed that PDGFRβ-expressing cells were located both on the scaffold surface and in the scaffold depths (Figure 4), whereas αSMA-expressing cells were mainly located around the vessel network in the scaffold depth. To assess the degree of cell proliferation at the various scaffold locations, samples were stained for Ki67. Ki67-expressing fibroblasts were significantly more frequent on the scaffold surface compared to the scaffold depth (Figure 5), suggesting that cell proliferation decreased within the deeper layers of the scaffold, closer to the forming vessels.

**Figure 6:**
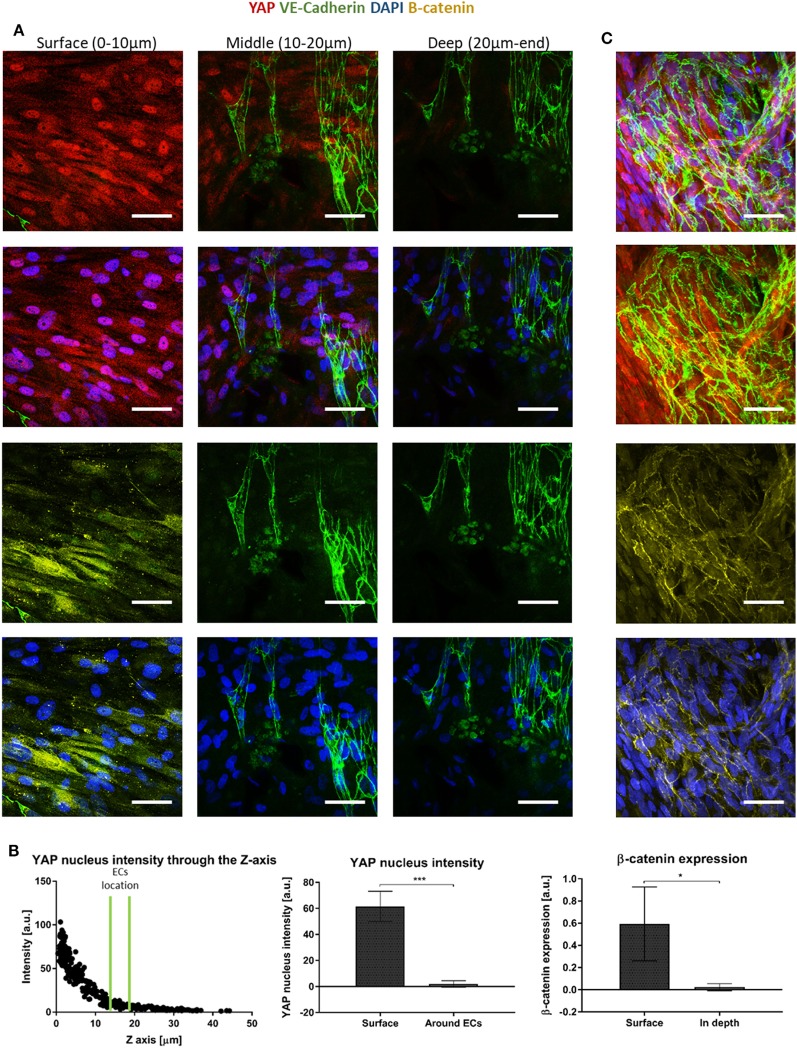
Yes-associated protein (YAP) and β-catenin expression across the scaffold. **(A)** Gelfoam scaffolds seeded with ECs and fibroblasts were stained for YAP (red), DAPI (blue), VE-cadherin (green), and β-catenin (yellow), 14 days after seeding. Various depths of the scaffolds were assessed for expression patterns. **(B)** Left—a representative graph of the distribution of nuclear YAP expression as a function of scaffold depth. EC location is marked by two green lines. Middle—quantification of nuclear YAP fluorescence intensity on the scaffold surface versus in areas in which ECs were observed. (*n* = 4) ***P-value <0.001. Right—quantification of β-catenin fluorescence intensity on the scaffold surface versus in the areas in which ECs were observed. (*n* = 3) *P-value <0.05. **(C)** ECs cultured for 4 days, at the surface of the scaffold formed sheets and expressed cytoplasmic β-catenin (yellow) and nuclear YAP (red). Scale bar indicates 50 µm. *P-value <0.05.

Since canonical Wnt signaling has been implicated in cell proliferation (Clevers, 2006), and is involved in supporting cell recruitment to forming vessel (Reis and Liebner, 2013), we next examined the expression patterns of proteins which play a central role in the Wnt canonical pathway within the cells composing the forming vessels. Cells grown on the scaffolds for 14 days were stained for YAP, β-catenin, and VE-cadherin. Localization of YAP in the fibroblast nucleus was only observed in the layers closer to the scaffold surface and its levels decreased with closer proximity to the scaffold core, where the endothelial cells were localized (Figure 6A). Fibroblasts β-catenin showed cytoplasmic localization, which also decreased in the scaffold depths (Figure 6B). For the cells grown for 4 days, cytoplasmic β-catenin and nuclear YAP were observed within the ECs sheets located on the surface of the scaffold, which formed at the earlier stages of culturing (Figure 6C).

### Fibroblast Proliferation Affects Vessel Formation

**Figure 7:**
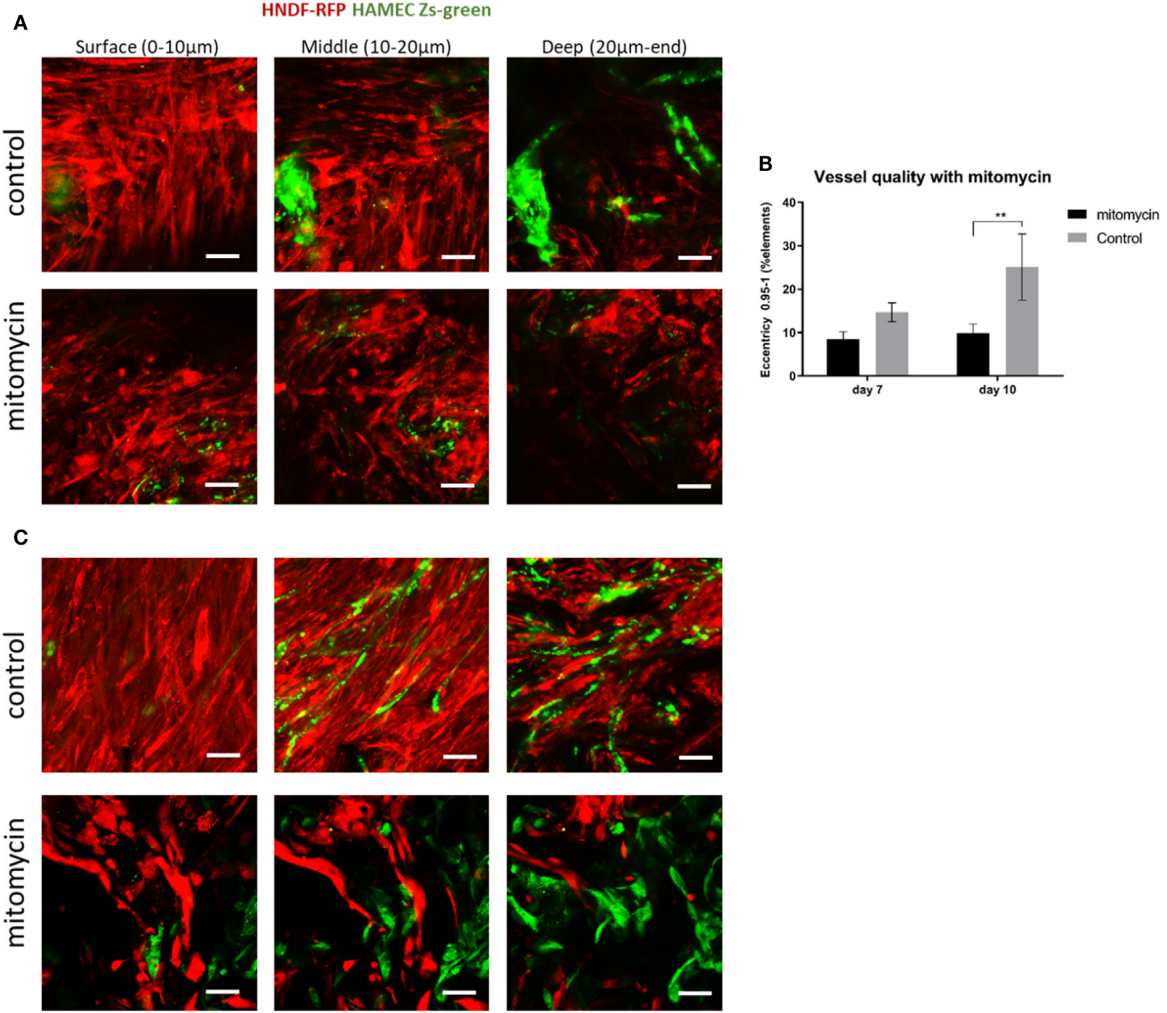
Mitomycin-treated fibroblasts impair blood vessel formation. Fibroblasts (red) treated with mitomycin were cocultured with ECs (green) on scaffolds, for 7 or 10 days. Images of mitomycin-treated and untreated scaffolds on **(A)** day 7 and **(B)** day 10 of culturing, at different depths of the scaffold. (*n* = 3) **P-value <0.01. **(C)** Quantification of vessel quality, assessed using the complexity parameter. Scale bar indicates 50 µm.

**Figure 8:**
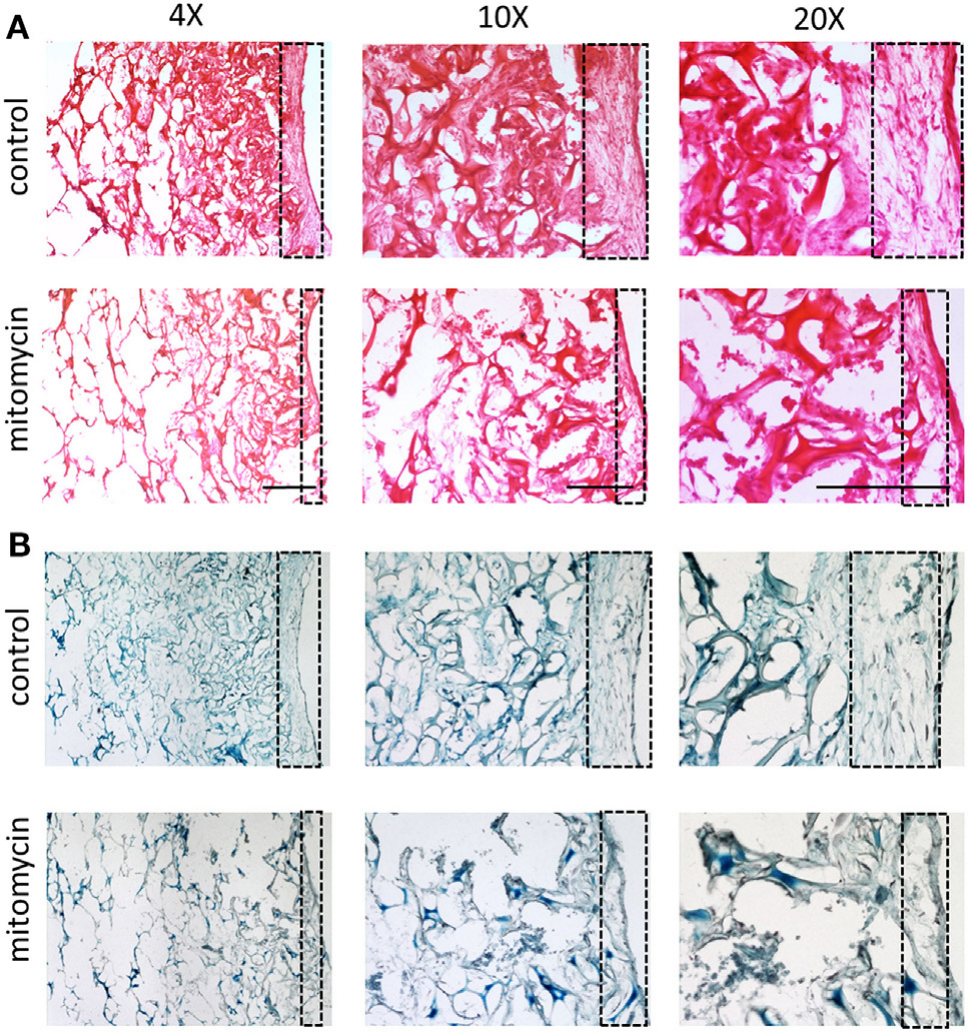
Cell layer and collagen density bearing mitomycin-treated fibroblasts. Scaffolds containing ECs and either untreated or mitomycin-treated fibroblasts, were cultured for 10 days. Transverse cryosections were then stained with **(A)** hematoxylin and eosin or **(B)** Masson’s trichrome. Dashed box indicates scaffold surface areas with dense tissue under normal conditions and thin tissue under mitomycin treatment. Scale bar indicates 400 µm.

The heightened expression of nuclear YAP and cytoplasmic β-catenin and more extensive cell proliferation on the scaffold surface, led us to investigate the role of Wnt signaling and specifically, of fibroblast proliferation in vasculogenesis. To this end, fibroblasts were treated with mitomycin prior to seeding and cell-embedded constructs were then grown for 7 or 10 days before being transversely cryosectioned. Confocal images of the various constructs revealed that the ECs seeded with mitomycin-treated fibroblasts, failed to form vessel networks (Figures 7A,B). In addition, the ECs in these constructs did not penetrate into the scaffold depths (Figure 7C), and remained localized in the same plane as the fibroblasts. Hematoxylin and eosin and trichrome stainings revealed a dense, thick layer of cells and collagen on the surface of scaffolds containing untreated fibroblasts, whereas the constructs with mitomycin-treated cells showed a thin layer, with less collagen deposition (Figures 8A,B, dashed box).

### ECs Seeding on Scaffolds Precultured with Fibroblasts

**Figure 9:**
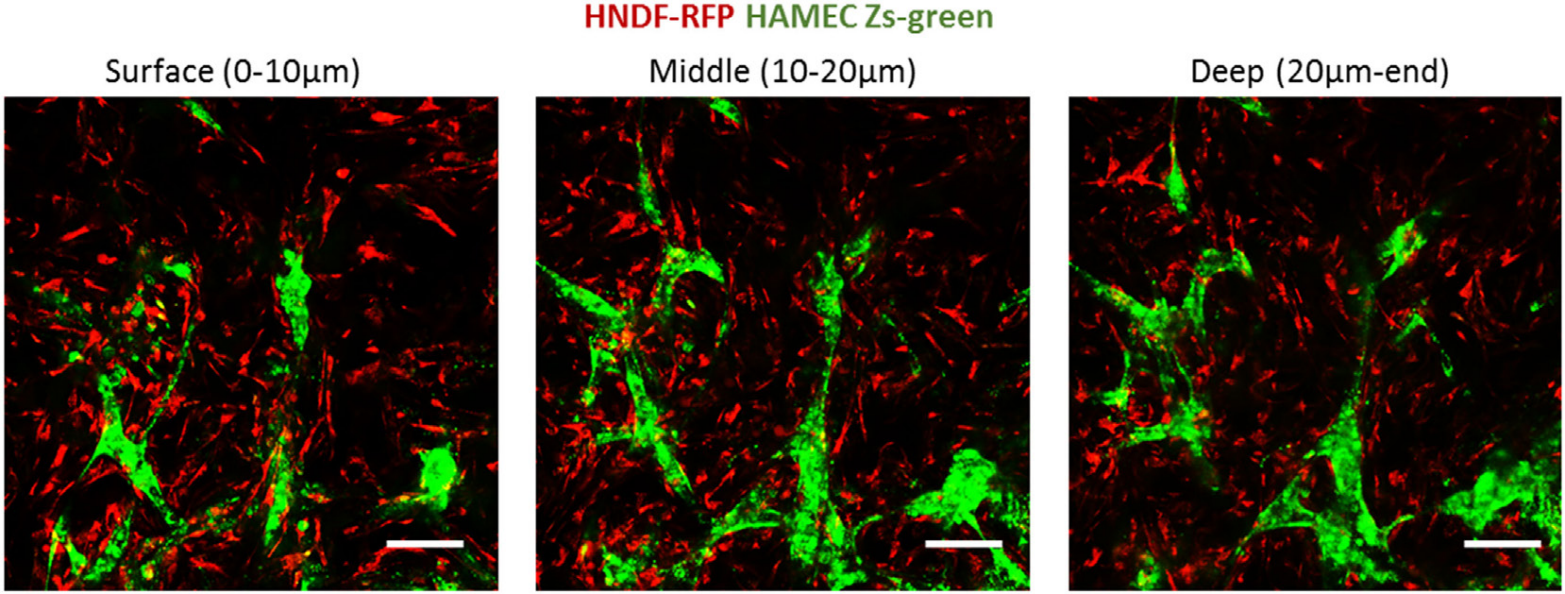
ECs penetration of a precultured fibroblasts layer. ECs (green) were seeded on scaffolds precultured with fibroblasts (red) for 12 days. Images were taken at the different depths of the scaffold, 5 days after reseeding. Scale bar indicates 50 µm.

Following the observed migration of ECs from the scaffold surface to its depth, when coseeded with fibroblasts, we set out to determine whether this migration occurs in the presence of a preexisting fibroblast layer. To this end, RFP-expressing HNDFs were seeded onto a gelfoam scaffold, and cultured for 12 days before Zs-green-expressing HAMECs were added to the culture. Despite the dense fibroblast layer, the endothelial cells migrated toward the inner part of the scaffold, where they formed a vessel network within 5 days (Figure 9). This migration might be due to the previously described hypoxic conditions in the scaffold depth.

## Discussion

To date, integration of engineered grafts into host tissue remains one of the main obstacles in tissue engineering. Growth of mature and functional blood vessels within the engineered constructs can overcome this problem, by ensuring graft perfusion, thereby preventing localized necrosis, until host-graft composite vessels are established (Shandalov et al., 2014). Biomimetic models designed to investigate vasculogenesis and angiogenesis processes (Nguyen et al., 2013; Landau et al., 2017; Perry et al., 2017) will ultimately empower construction of improved blood vessel networks.

In this study, tissue engineering techniques were used as a platform to investigate blood vessel development and to investigate cell properties throughout 3D environments of vascular networks. For this purpose, a combination of ECs and fibroblasts or DPSCs was seeded into both PLLA-PLGA scaffolds and gelfoam gelatin sponges. The cell combination formed vessels in a spatially dependent manner, with dense layers of fibroblasts in the surface area and EC-rich vessels, with a thin enveloping fibroblast layer, in the depths of the scaffold. Moreover, the cells showed location-distinct characteristics across the scaffold, with the fibroblasts on the surface appearing to be more proliferative, and expressing more nuclear YAP, while those surrounding the vessels deep within the scaffold, expressed more αSMA.

Mural pericytes impart a nascent vessel stabilizing effect, *via* a process mediated by various cytokines such as, TGF-β1, which promotes mesenchymal precursor cells differentiation into pericytes, and EC-derived platelet-derived growth factor subunit B (PDGF-B), which recruits PDGFR-β-expressing pericytes, which, in turn, migrate toward the forming vessel and surround it (Welti et al., 2013). In the present model, PDGFR-β expression was observed in cells on the scaffold surface and in the cells surrounding the vessels deep within the scaffold, leading us to conclude that PDGFR-β-expressing cells are located all over the scaffolds and are being recruited toward the nascent vessels. In contrast, αSMA expression in vessel supporting cells was rarely detected in cells at the scaffold surface, and distinctly localized around mature vessels, likely mechanically stabilizing the forming vessel. This observation is in keeping with reports of mechanical contractile forces on vessels induced by pericytes enveloping vessels (Bergers and Song, 2005; Volz et al., 2015).

Wnt has been shown to play a significant role in vascular morphogenesis and to promote cell proliferation. When the Wnt pathway is active, cytoplasmic YAP is inhibited, resulting in its accumulation in the nucleus, which, in turn, leads to stabilization of β-catenin in the cell cytoplasm. In contrast, in the absence of Wnt signaling, YAP/TAZ takes part in a destruction complex that degrades β-catenin (Cheng et al., 2003; Azzolin et al., 2014). Thus, we hypothesized that the observed accumulation of nuclear YAP and cytoplasmic β-catenin in the cells located on the scaffold surface, were in a “Wnt ON” state, which could explain the high proliferation rate recorded at the scaffold surface. In contrast, the cells in the inner part of the scaffolds were likely in a “Wnt OFF” state, with lower nuclear YAP and β-catenin levels. When inhibiting fibroblast proliferation, blood vessels failed to form. Thus, we hypothesize that the excessive proliferation of the supporting cells at the scaffold surface, which leads to higher production of ECM, and higher secretion of proangiogenic factors is a prerequisite for blood vessel formation and stabilization.

Additionally, the cells forming the endothelial sheets appeared to express nuclear YAP and cytoplasmic β-catenin. The wnt/β-catenin pathway has been shown to enhance the recruitment of mural cells by PDGF-B and to stabilize the forming vessel (Reis and Liebner, 2013). Thus, we speculate that the endothelial sheets recruit the supporting cells, which then provide mechanical support (Bergers and Song, 2005) and proangiogenic signals (Welti et al., 2013), enabling formation of blood vessels.

The presented model is unique in its reproducibility, independent of the specific cell types or the biomaterials that were used. Coseeding endothelial cells and supporting cells, from different primary cells, within supportive 3D environments allows for the formation of vessel-like structures. The culture conditions for vessel formation, such as cell ratios and media content, may vary according to the specific cell type and specific donor. However, once the vessel network is formed, endothelial cell migration into the scaffold depth and proliferation of supporting cells on the scaffold surface were detected. Though, we cannot exclude that altering the experiment design and the cell type will result in a different phenomenon. The ability to track these processes within 3D environments *in vitro* will enable us to further characterize location-specific cell characteristics.

## Author Contributions

ShiL performed the research; ShiL, SG, and ShuL designed the research, analyzed the data, and wrote the article.

## Conflict of Interest Statement

The authors declare that the research was conducted in the absence of any commercial or financial relationships that could be construed as a potential conflict of interest. The reviewer AS and handling Editor declared their shared affiliation.

## Supplementary Material

Figure S1 NG2 and VWF staining of vessel constructs. ECs and fibroblasts were seeded into a gelfoam scaffold, fixed and then stained for vWF (red, for ECs) and NG2 (green, for fibroblasts). Images present the endothelial cell and fibroblast morphology at different depths of the scaffold on day 14 of culturing.

Figure S2 Lumenized vascular networks. 3D confocal imaging show lumen formation within the vessels, ZY and ZX are transverse views of the vessels.
