# Supplementary figures and images for: Localization of Engineered Vasculature within 3D Tissue Constructs
Source: Front Bioeng Biotechnol. 2018 Jan 22;6:2. doi: 10.3389/fbioe.2018.00002 (PMC5786867; doi:10.3389/fbioe.2018.00002)

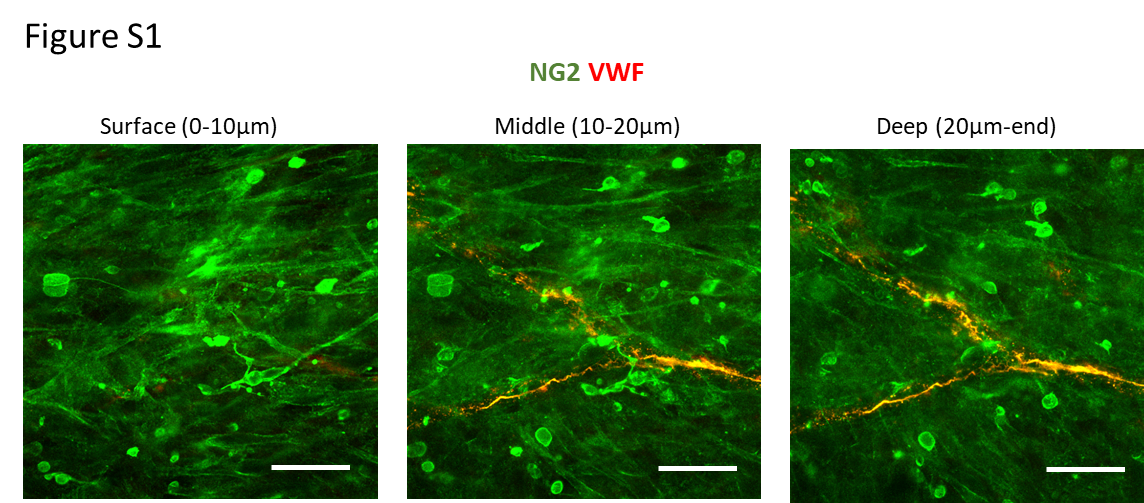

Supplement: Figure S1 — NG2 and VWF staining of vessel constructs. ECs and fibroblasts were seeded into a gelfoam scaffold, fixed and then stained for vWF (red, for ECs) and NG2 (green, for fibroblasts). Images present the endothelial cell and fibroblast morphology at different depths of the scaffold on day 14 of culturing. [file Image_1.tif]

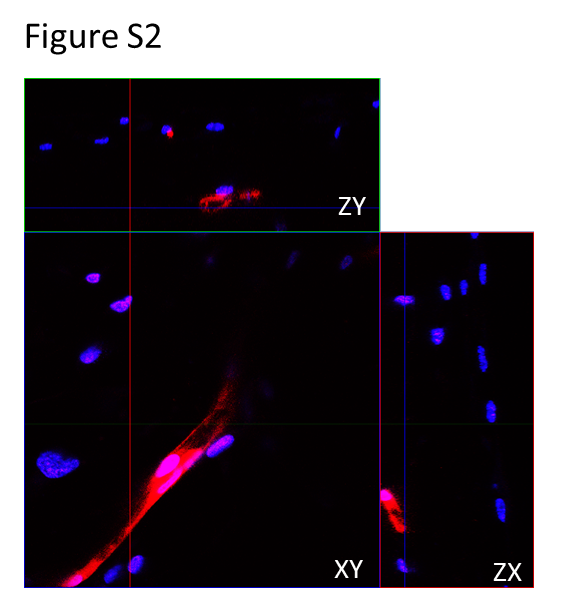

Supplement: Figure S2 — Lumenized vascular networks. 3D confocal imaging show lumen formation within the vessels, ZY and ZX are transverse views of the vessels. [file Image_2.tif]
